# Supplementary material for: Splice-Junction-Based Mapping of Alternative Isoforms in the Human Proteome
Source: Cell Rep. Author manuscript; Available in PMC 2020 Jan 15. (PMC6961840; doi:10.1016/j.celrep.2019.11.026)
Supplement: 3 [file NIHMS1546469-supplement-3.zip › DF2/PXD0010154/Liver-119-Q86SQ0-TKDADLLDVESK.pdf]

A

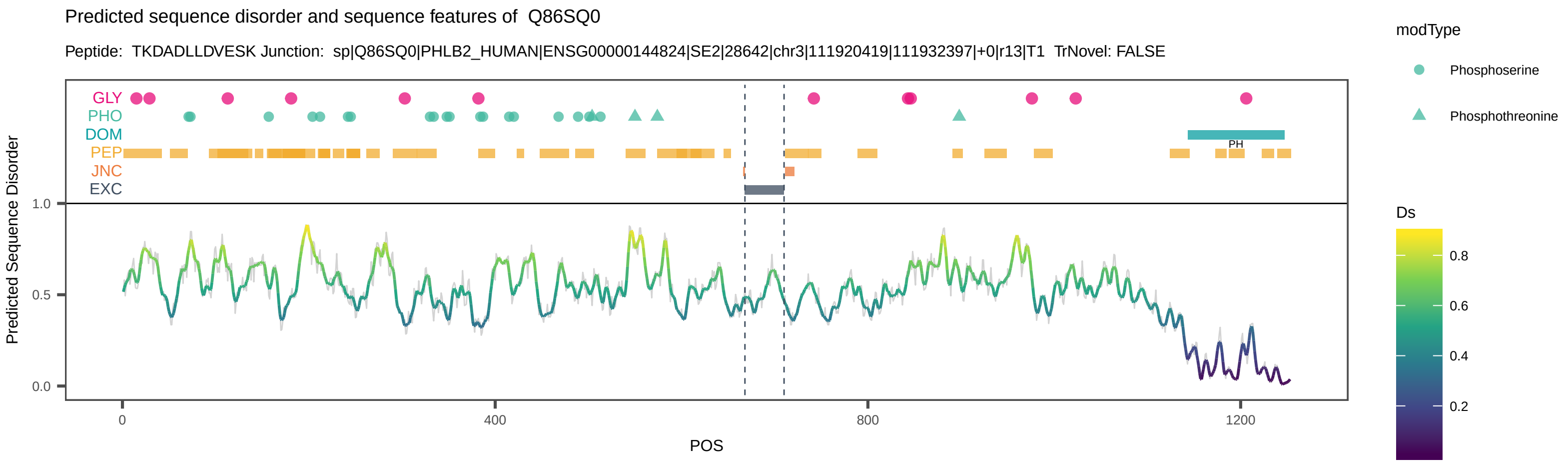

Predicted sequence disorder and sequence features of Q86SQ0
Peptide: TKDADLLDVESK Junction: sp|Q86SQ0|PHLB2_HUMAN|ENSG00000144824|SE2|28642|chr3|111920419|111932397|+0|r13|T1 TrNovel: FALSE
GLY
PHO
DOM
PEP
JNC
EXC
PH
Predicted Sequence Disorder
1.0
0.5
0.0
0
400
800
1200
POS
modType
Phosphoserine
Phosphothreonine
Ds
0.8
0.6
0.4
0.2

B

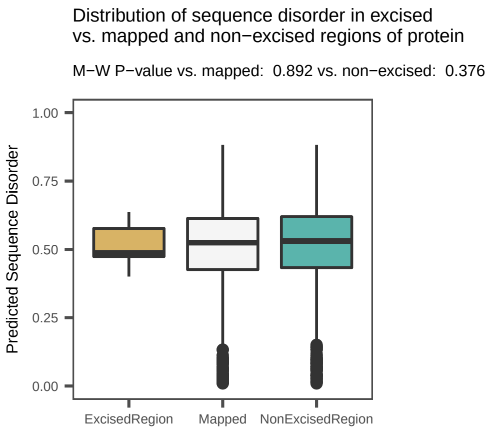

Distribution of sequence disorder in excised
vs. mapped and non−excised regions of protein
M−W P−value vs. mapped: 0.892 vs. non−excised: 0.376
Predicted Sequence Disorder
1.00
0.75
0.50
0.25
0.00
ExcisedRegion
Mapped
NonExcisedRegion

C

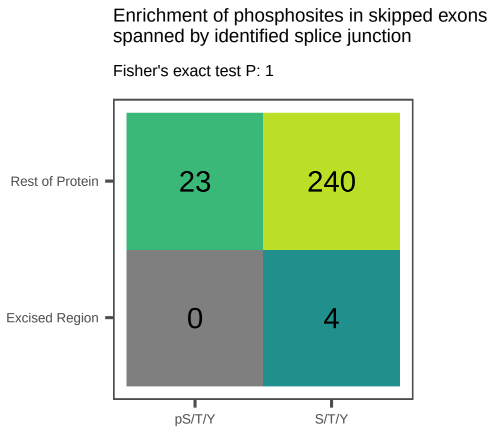

Enrichment of phosphosites in skipped exons
spanned by identified splice junction
Fisher's exact test P: 1
Rest of Protein
Excised Region
23
240
0
4
pS/T/Y
S/T/Y
